# Experiences of patients with poststroke spasticity throughout a botulinum toxin treatment cycle: Results from a prospective ethnographic study

**DOI:** 10.3389/fneur.2022.946500

**Published:** 2022-08-23

**Authors:** Jorge Jacinto, Andreas Lysandropoulos, Marjorie Leclerc, Françoise Calvi-Gries

**Affiliations:** ^1^Alcoitão Rehabilitation Medicine Center, Estoril, Portugal; ^2^Ipsen, Cambridge, MA, United States; ^3^Cerner Enviza, Paris, France; ^4^Ipsen, Boulogne, France

**Keywords:** poststroke spasticity, botulinum toxin, ethnographic study, patient-reported outcomes, semi-structured qualitative study

## Abstract

This study was conducted to capture the experience of patients with poststroke spasticity (PSS) throughout one botulinum neurotoxin A (BoNT-A) treatment cycle. The REBOT study (NCT03995524) was a prospective, observational ethnographic study conducted in France, Italy, the UK, and the USA. It combined a mixed-method ethnography (including semi-structured qualitative interviews within a week of a BoNT-A injection) with completion of a longitudinal quantitative patient-reported outcome questionnaire and sharing of video and images, both reported weekly over a 12–14-week period throughout the BoNT-A treatment cycle. The study recruited 30 adult patients with PSS who were receiving BoNT-A treatment. The most commonly used BoNT-A product was onabotulinumtoxinA (Botox^®^), which was administered to 21 patients (70%), whereas two patients (6.7%) received abobotulinumtoxinA (Dysport^®^) and seven patients (23.3%) did not specify the BoNT-A medication that they received. Patients reported a high, continuous burden of PSS, with spasms, sleeping difficulties, stiffness, and pain being the most commonly reported symptoms. In line with an observed waning effect of BoNT-A injections, spasticity symptoms initially were improved at Weeks 4–6 after injection but reemerged after 9–11 weeks. Treatment satisfaction levels decreased over the BoNT-A treatment cycle, as reflected by the worsening of symptoms and the need to self-medicate and consult a physician. The psychological impact of PSS was high. Patients acknowledged the benefits of BoNT-A treatment but wished for more individualized treatment plans with flexible dosing and injection intervals. Additionally, only 10% of patients reported that they had a trusting relationship with their physician and believed that their needs were considered by those managing their PSS. To our knowledge, this was the first ethnographic study in patients with PSS who were treated with BoNT-A. This ethnographic approach to patient surveys complements traditional research methods and allows improved identification of patients' unmet needs by capturing their weekly experience of treatment. The findings of this study confirm previous observations of the diminishing effectiveness of BoNT-A injections between treatment sessions, highlighting the need for agents with a longer duration of action and/or a more flexible treatment pattern that allows for more frequent injections.

## Introduction

Despite improvements in outcomes in the past decade, stroke remains the second leading cause of death worldwide and is a major cause of disability and morbidity in survivors. It is estimated that a quarter of people aged 25 years or older will have a stroke during their lifetime (1). A global systematic analysis of disease burden revealed that, in 2016, there were more than 80 million stroke survivors and stroke constituted 42.2% of all global neurological disability-adjusted life-years (2, 3). Spasticity is defined as a sensorimotor disorder, resulting from an upper motor neuron lesion, that presents as intermittent or sustained involuntary activation of muscles (4).

Up to 40% of stroke survivors develop spasticity, with the initial development of paresis being an important predictor of spasticity over the long term (5). Poststroke spasticity (PSS) is typically a chronic condition that has a significant impact on patients' health-related quality of life (QoL) (6). Regardless of etiology, spasticity affects patients' functional ability, activities of daily living, independence, social life, and work life, and so may incur a significant burden to their caregivers (7, 8).

The goal of PSS management is to facilitate patients' neurological recovery and improve symptom control as well as active and passive functions of the affected limbs (6, 9). Management of PSS should be provided within the context of a wider rehabilitation program and involve a range of interventions tailored to patient needs (10). These interventions may include physical therapy, occupational therapy, orthotics, oral antispasmodic medication, or local injections with botulinum neurotoxin A (BoNT-A) (6).

In combination with rehabilitation, BoNT-A has become a standard of care in PSS. BoNT-A treatment provides a sustained reduction in muscle tone, which translates into symptom control and improvements in active and passive function outcomes, tolerance/endurance, compliance to other therapies, caregiver burden, and overall health-related QoL in terms of the symptoms of the upper and lower limbs (6, 11–13).

Although BoNT-A is an acknowledged pharmacological treatment for spasticity (6, 14), the effects of BoNT-A are not permanent and further injections are likely to be required every 3–4 months (6). In addition, the reemergence of symptoms between two planned treatment sessions is common, necessitating an increased frequency of injections (7, 8).

This waning effect of BoNT-A has been associated with increased cost and patient dissatisfaction with treatment (7, 8). A clear understanding by clinicians of the patients' experiences is important for providing effective individualized care, yet there remains a knowledge gap regarding how recurring symptoms may affect patients' daily lives.

Ethnographic surveys are relatively new in clinical studies; to date, only a few studies in stroke survivors have used this approach to understand patients' experiences (15, 16). To our knowledge, this method has not yet been used specifically in patients with spasticity of any etiology. In ethnography, observation takes place in the participants' day-to-day setting, allowing researchers to understand how an event is perceived and interpreted in a real-world situation (17).

Our study was designed to use an ethnographic approach to address the gap in our understanding of the daily impact of symptom reemergence in patients living with PSS.

## Materials and methods

### Study design

The REBOT study (NCT03995524) was a prospective, observational ethnographic study conducted in France, Italy, the UK, and the USA. This study combined a mixed qualitative approach, using interviews and video and/or image sharing, with quantitative patient-reported outcome (PRO) measures to directly capture the experiences of patients with PSS throughout one BoNT-A treatment cycle.

**Figure 1:**
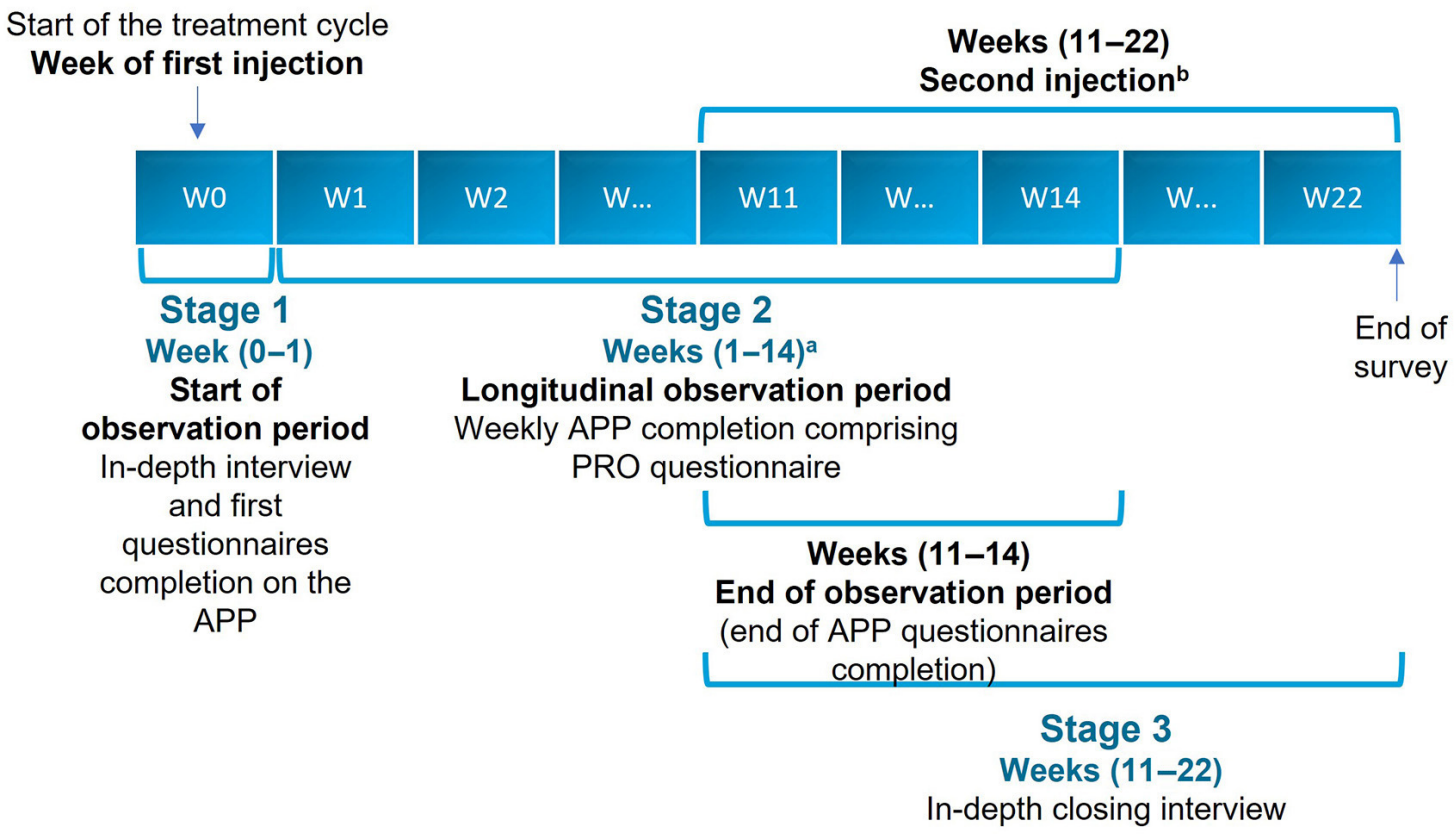
Study flowchart. ^a^Weekly completion of all sections of the APP, except the WHODAS 2.0, which was completed monthly. ^b^Except for two patients who did not receive their second injection by Week 22. APP, application; PRO, patient-reported outcome; W, week.

The study was conducted in three stages (Figure 1).

#### Stage 1

In-depth, qualitative, face-to-face interviews or web-assisted telephone interviews were conducted up to 1 week after BoNT-A injection to describe the patients' experience of spasticity and their perception of disease burden and treatment.

#### Stage 2

Longitudinal ethnographic observation using a quantitative PRO questionnaire was delivered *via* a smartphone/tablet application developed for the study, which contained a questionnaire with *ad hoc* questions. This tool also invited patients to undertake tasks, which included submitting videos and photographs as well as images from the internet. The observation period for each patient started at baseline, which was set no later than 1 week after their BoNT-A injection. The longitudinal observation phase lasted for 12–14 weeks. This stage was conducted to assess weekly changes in patients' self-reported outcome parameters.

- The PRO questionnaire comprised the 5-level, 5-dimension EuroQol questionnaire (EQ-5D-5L), the World Health Organization Disability Assessment Schedule 2.0 (WHODAS 2.0) and a series of *ad hoc* questions, including the impact of symptoms on mobility, mood, and treatment satisfaction.
- During this longitudinal phase, patients were encouraged to share images, videos, and audio recordings to complement and contextualize the data; this stage used personal accounts to capture the patients' emotional states and the impact of the spasticity on their daily life.
- To capture dynamic changes in symptom burden, four main time points were established: baseline, between Weeks 4 and 6, between Weeks 9 and 11, and the end of the observation period. In this schedule, Week 0 relates to the injection date, and Week 1 relates to the injection date +7 days.

#### Stage 3

In-depth, qualitative, face-to-face interviews or web-assisted telephone interviews were undertaken to review and take stock of the BoNT-A treatment course experience. This closing interview was scheduled to be undertaken at approximately 12 weeks and took place no later than 2 weeks after the second injection and no later than 22 weeks after the first injection.

### Inclusion criteria

The study included adults aged 18–75 years who had received a diagnosis of PSS more than 3 months before inclusion, were ambulatory, were able to give informed consent for participation, and had access to and the ability to use a smartphone or tablet.

Patients were required to have been receiving BoNT-A injections for PSS for at least 6 months (or had received two cycles of treatment), with BoNT-A injection cycles lasting for no more than 16 weeks.

### Exclusion criteria

Patients were excluded from the study if they had one or more of the following:

- physical disability due to reasons other than stroke
- neurolysis or surgery to the affected limb in the 6 months before screening
- changes to their spasticity-specific treatment in the 3 months before screening
- simultaneous participation in a clinical trial for the treatment of spasticity.

### Treatment

This was a non-interventional study and BoNT-A treatment was prescribed to patients with PSS before, and independently of, enrollment in the study.

### Study objectives

The primary objective of this observational ethnographic study was to investigate the changes in daily symptom burden and disability in patients with PSS, capturing the effects of these symptoms on the patient's ability to function and to perform daily tasks, and on their health-related QoL throughout one BoNT-A treatment cycle.

The secondary objectives were to:

- identify domains related to the everyday life experiences (such as the burden of spasticity symptoms and treatment) of patients with PSS through qualitative analysis of in-depth, open-ended interviews
- characterize patients' satisfaction over the BoNT-A treatment cycle and their desire for tailored treatment options.

See the Supplementary material for examples of the types of collected data.

### Data analysis

NVivo 12 Plus software was used for coding, linking, and retrieving the qualitative data from the interview transcripts. Rating scales from the questionnaire included validated PRO material, which was analyzed using SPSS Statistics version 22, Stata version 15, Microsoft Excel version 15, and Microsoft Power BI according to the type of analysis and nature of the data. The normality of data distribution was assessed using histograms, Q-Q plots and P-P plots. Planned analyses included, as appropriate, the Student's paired *t*-test or Wilcoxon signed-rank test. All statistical tests were two-sided to determine significance at the 5% level. EQ-5D was converted into a health utility score using each country's value set. EQ-5D items, utility score, EuroQol visual analog scale (EQ-VAS) as well as WHODAS items and mean scores were analyzed descriptively, including 95% confidence intervals. EQ-5D-5L utility score and EQ-VAS between the beginning of the observation and Weeks 4–6 were compared (Student's paired *t*-test). WHODAS mean scores between the beginning of the observation and Week 5 were compared. To handle missing data, we used multiple imputations for EQ-5D-5L and a mean of available items for WHODAS 2.0 when only one item is missing a value.

Videos and images were transcribed into text and coded with Excel based on a common codeframe. To ensure comparability of data collected from different sources (i.e., audio-taped interviews, rating scales, photos, and videos), a structured framework approach was used initially to group findings into descriptive codes, which were later sorted into categories and interpretive themes.

## Results

### Study participants

Patient demographics and baseline characteristics are summarized in Table 1. The study included 30 patients. In all, 21 patients (70%) were women, the mean patient age was 48.7 years (standard deviation [SD]: 12.7 years) and 24 patients (80%) were younger than 60 years.

**Table 1:** Patient baseline demographics and characteristics.

Age groups (years)	
< 30	2 (6.7)
30–45	10 (33.3)
46–59	12 (40.0)
≥ 60	6 (20.0)
Mean age (SD)	48.7 (12.7)
**Gender**	
Male	9 (30)
Female	21 (70)
**Country**	
France	9 (30.0)
Italy	2 (6.7)
UK	7 (23.3)
USA	12 (40.0)
**Time from spasticity diagnosis (years)**	
< 3	7 (23.3)
3–6	11 (36.7)
> 6	12 (40.0)
**Neurotoxin brand used**	
Botox^®^	21 (70.0)
Dysport^®^	2 (6.7)
Unspecified BoNT-A	7 (23.3)
**Concomitant therapy** ^a^	
None	4 (13.3)
Received a concomitant treatment at enrollment	17 (56.7%)
Physiotherapy/kinesitherapy/balneotherapy	10 (33.3)
Baclofen	5 (16.6)
Clonazepam and amitriptyline	1 (3.3)
Dantrium	1 (3.3)
Oxazepam and ergotherapy	1 (3.3)
Tricidine	1 (3.3)
Unspecified	2 (6.7)

All values are N (%) unless otherwise specified.

a Two patients had undergone surgery within the permitted timeframe before study initiation (> 6 months); no changes in spasticity-specific treatment were allowed within 3 months before inclusion. BoNT-A, botulinum neurotoxin A; SD, standard deviation.

The most commonly used BoNT-A product was onabotulinumtoxinA (Botox^®^), used by 21 patients (70%). A further two patients (6.7%) received abobotulinumtoxinA (Dysport^®^) and seven patients (23.3%) did not specify the BoNT-A medication that they received.

### Concomitant therapy

Overall, 26 patients (86.7%) received previous treatment before enrollment and 17 (56.7%) received concomitant treatment. Among all patients, 10 (33.3%) underwent concomitant physiotherapy and/or kinesitherapy and/or balneotherapy and the main concomitant medication was baclofen, used by five patients (16.6%).

### Primary analysis

The primary analysis was based on data from stage 2 of the study, including data from the quantitative PRO questionnaire.

#### Changes in symptom burden and disability across a BoNT-A treatment cycle

**Figure 2:**
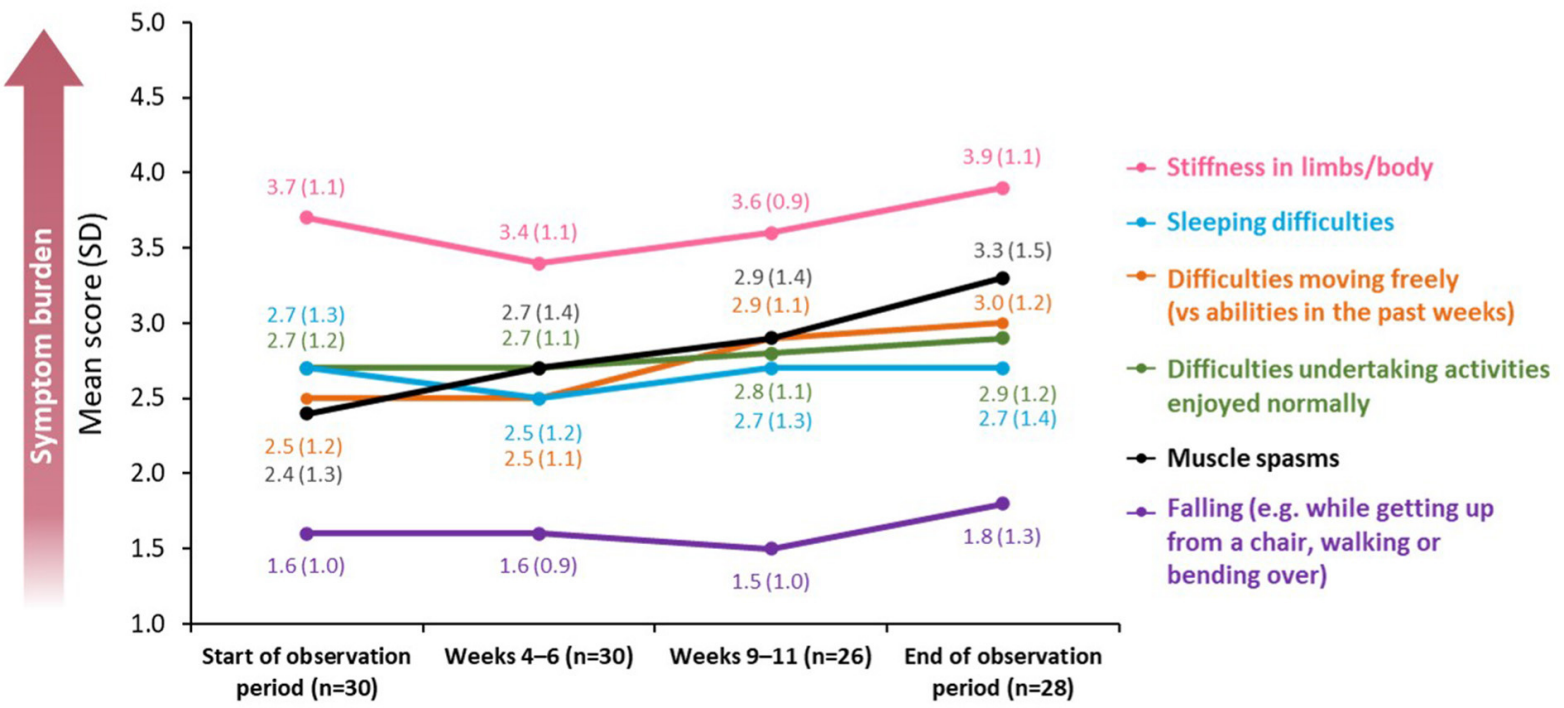
Changes in symptom burden and disability across a BoNT-A treatment cycle. BoNT-A, botulinum neurotoxin A; SD, standard deviation.

During the study, patients rated their symptom burden using a five-point scale, in which 1 = “symptoms do not occur at all” and 5 = “symptoms occur very often” (Figure 2).

Stiffness in limbs and sleeping difficulties were reported as the most burdensome symptoms (mean score at baseline of 3.7 [SD: 1.1] and 2.7 [SD: 1.3], respectively; *n* = 30). For both measures, there were improvements following treatment, with a mean decrease of 0.3 and 0.2 points, respectively, at peak effect in Weeks 4–6 (*n* = 30) before levels returned to baseline in Weeks 9–11 (*n* = 26).

Difficulties in moving freely remained the same from baseline until Weeks 4–6 [mean score of 2.5 (SD: 1.2) and 2.5 (SD: 1.1), respectively; *n* = 30] but worsened by the end of the observation period [mean score of 3.0 (SD: 1.2); *n* = 28]. A similar trend was observed for difficulties in undertaking activities that the patient would normally enjoy [mean score 2.7 at baseline and at Weeks 4–6 (SD 1.2 and 1.1, respectively; *n* = 30) rising to 2.9 (SD: 1.2; *n* = 28) at the end of the observation period].

The burden of falling was minimal throughout the observation period [mean score of 1.6 (SD: 1.0; *n* = 30) at baseline vs. 1.8 (SD: 1.3; *n* = 28) at the end of the observation period].

Across all affected domains, the only significant difference in mean score between baseline and the end of the observation period was recorded for muscle spasms, which increased between the two time points [mean score change of 0.82 (SD: 1.28), *p* = 0.033; *n* = 28].

#### Need for symptom alleviation

**Figure 3:**
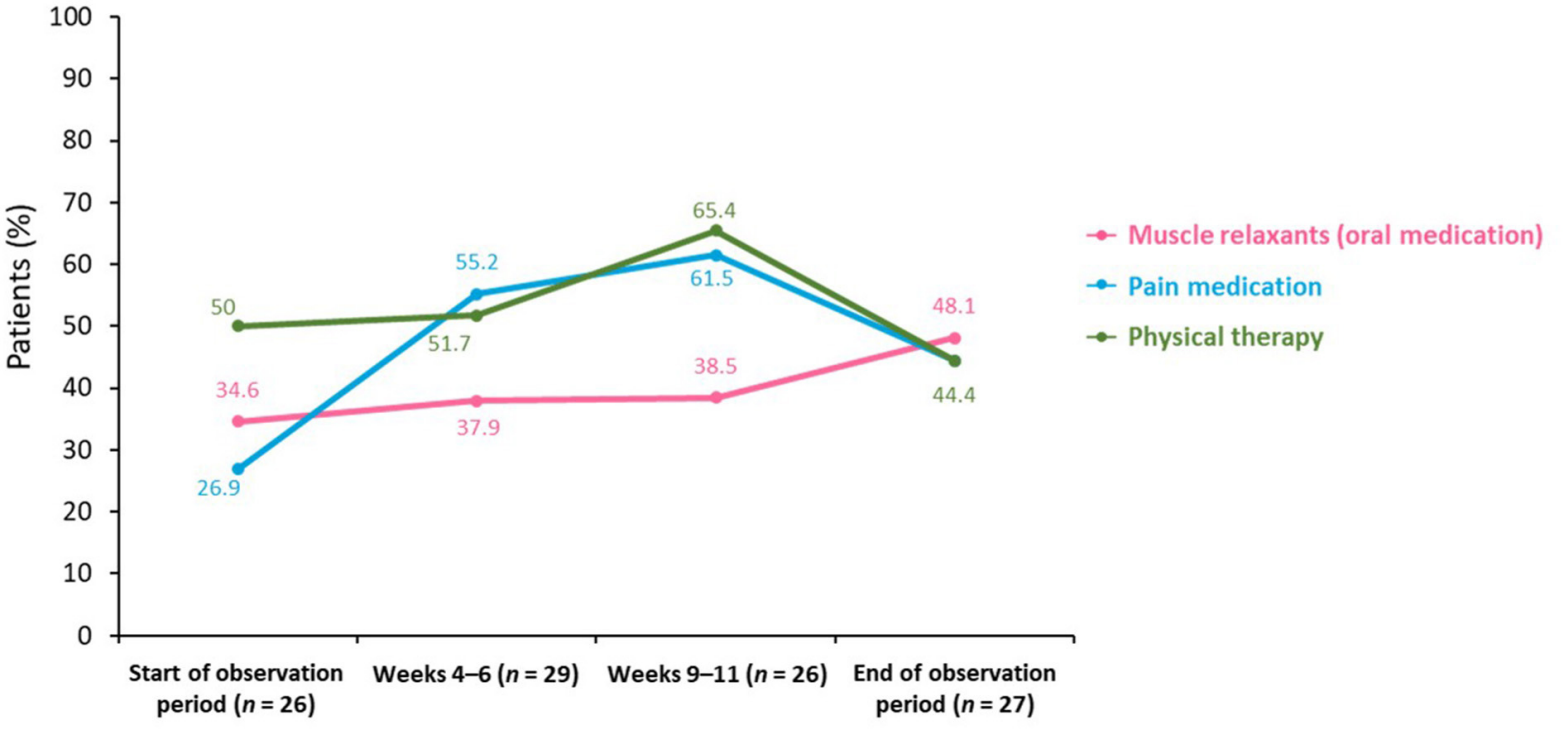
Need for self-medication across a BoNT-A treatment cycle. BoNT-A, botulinum neurotoxin A.

Patients' self-medication to alleviate symptoms depended on their selected approach (Figure 3). The proportion of patients using muscle relaxants increased from 34.6% at the start of the observation period (*n* = 9) to 48.1% at the end of the observation period (*n* = 13).

The proportion of patients using pain medication increased markedly between the start of the observation period and Weeks 4–6 [from 26.9% (*n* = 7) to 55.2% (*n* = 16)] before peaking at Weeks 9–11 (61.5%; *n* = 16). At the end of the observation period, the proportion decreased to 44.4% (*n* = 12).

The proportion of patients undergoing physical therapy was highest at Weeks 9–11 (65.4%; *n* = 17). More patients wanted to see their neurologist or treating specialist at the end of the observation period (48.1%; *n* = 13) than at the beginning (15.4%; *n* = 4).

#### Overall weekly mood and treatment satisfaction

The study also assessed overall weekly mood compared with each previous week in addition to satisfaction with the effect of the last BoNT-A injection. These items were evaluated using a five-point scale on which 1 = “very dissatisfied” and 5 = “completely satisfied.”

**Figure 4:**
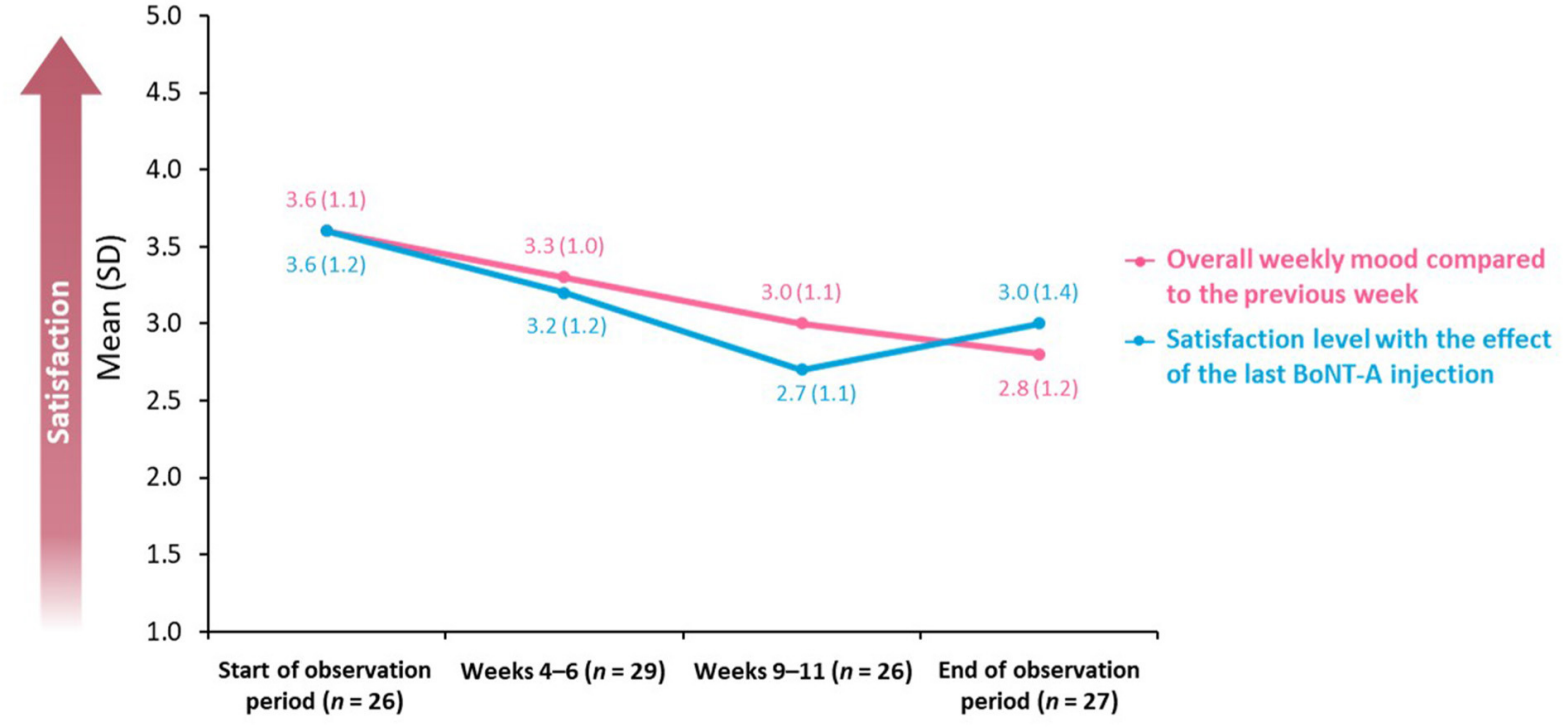
Overall weekly mood and BoNT-A treatment satisfaction. BoNT-A, botulinum neurotoxin A; SD, standard deviation.

The mean score for overall weekly mood decreased from 3.6 (SD: 1.1; *n* = 26) to 2.8 (SD: 1.2; *n* = 27) during the observation period (Figure 4). Similarly, the level of satisfaction with the effect of the last BoNT-A injection decreased between the beginning of the observation period [mean score of 3.6 (SD: 1.2); *n* = 26] and Weeks 9–11 [mean score of 2.7 (SD: 1.1); *n* = 26].

Patients submitted photographs of their affected limbs at different stages of stiffness and shared images that represented highlights and low points in terms of their mood. Chosen themes of the highlight images included hope, joy, being outside, and meeting family, whereas the low-point images included anger or frustration, pain, and stiffness.

#### QoL and mobility assessments

##### EQ-5D-5L

The EQ-5D-5L uses a five-point scale (on which 1 = “no problems” and 5 = “unable to/extreme problems”) and was used to measure the impact of spasticity on mobility, self-care, usual activities, pain/discomfort, and anxiety/depression.

**Figure 5:**
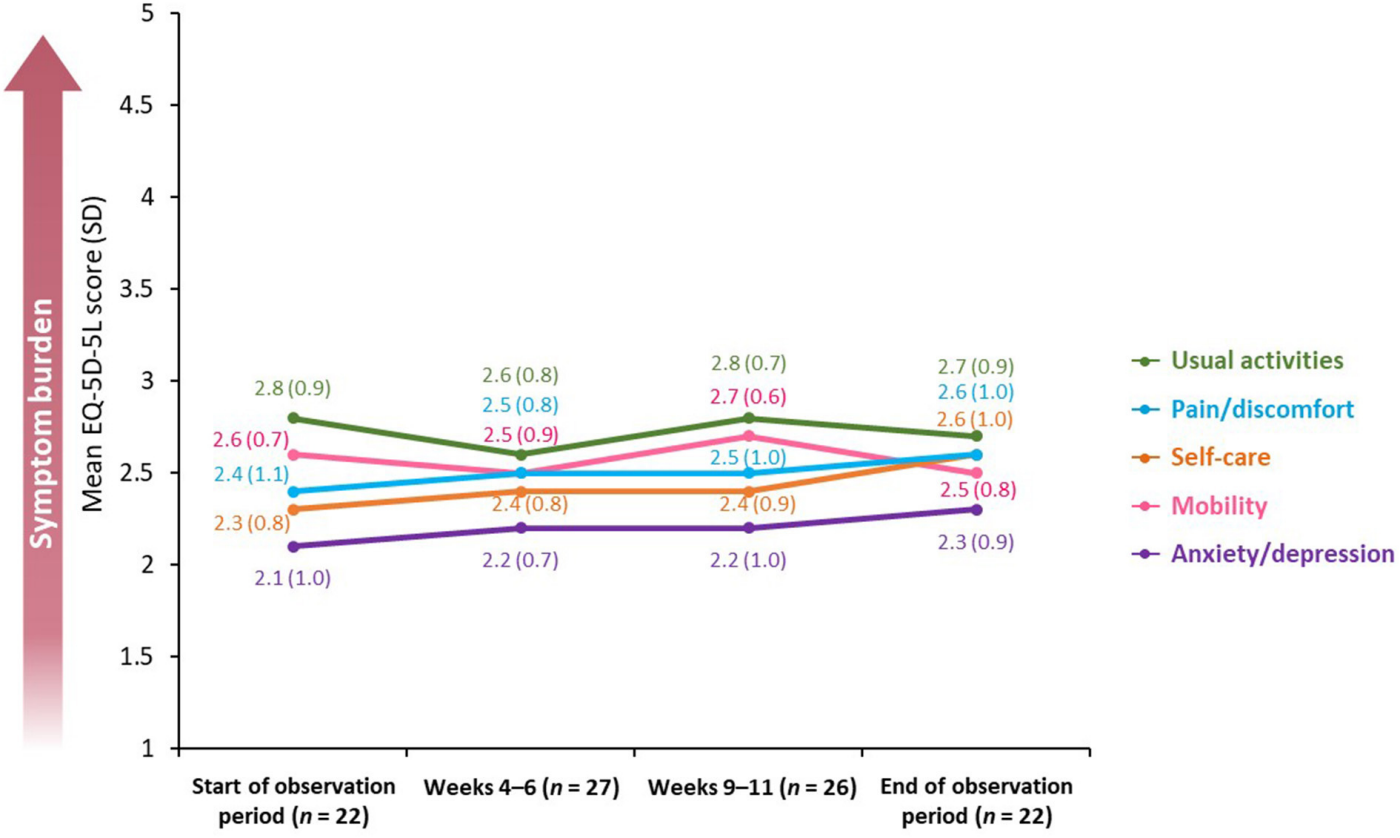
Mean EQ-5D-5L score across a BoNT-A treatment cycle. BoNT-A, botulinum neurotoxin A; EQ-5D-5L, 5-level, 5-dimension EuroQol questionnaire; SD, standard deviation.

Overall, the burden of symptoms was relatively stable over the observation period [mean difference between the beginning and end of the observation period in EQ-5D-5L utility score was 0.04 (SD 0.2; *n* = 30; *p* = 0.5)]; nevertheless, EQ-5D-5L mobility and usual activities scores were affected the most, peaking at Weeks 9–11 [mean scores of 2.7 (SD: 0.6; *n* = 26) and 2.8 (SD: 0.7; *n* = 26), respectively] (Figure 5).

##### WHODAS 2.0

**Figure 6:**
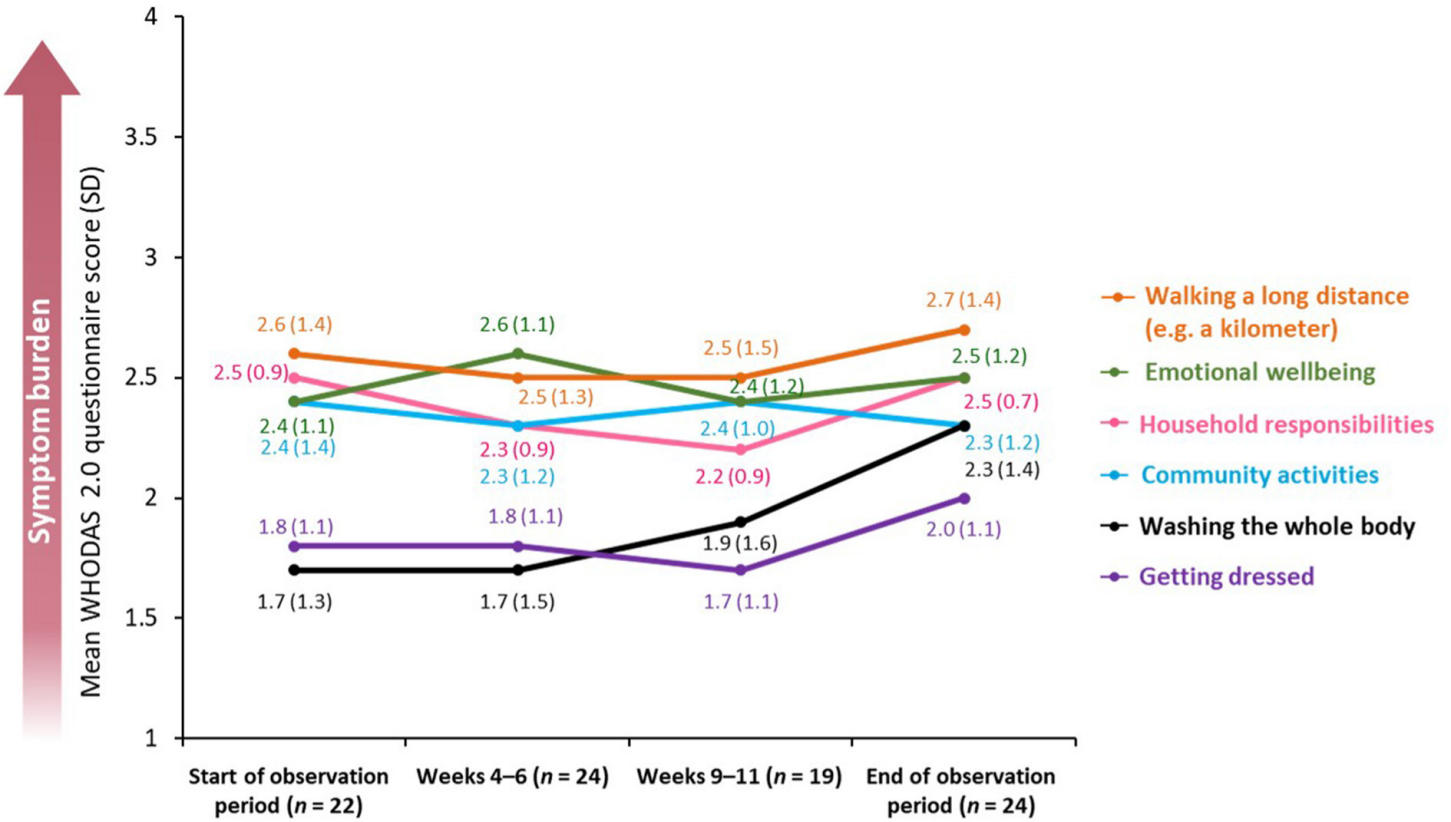
Mean WHODAS 2.0 questionnaire score across a BoNT-A treatment cycle. BoNT-A, botulinum neurotoxin A; WHODAS 2.0, World Health Organization Disability Assessment Schedule 2.0; SD, standard deviation.

Similarly to the EQ-5D-5L, results obtained using the WHODAS 2.0 questionnaire revealed that, overall, symptom burden was stable throughout the observation period. The biggest reported difference between the beginning and the end of the observation period was in the ability of patients to wash their whole body [change in mean score from 1.7 (SD: 1.3; *n* = 22) to 2.3 (SD: 1.4; *n* = 24) using a five-point scale on which 0 = “no difficulty” and 4 = “extreme difficulty”) (Figure 6). There was no significant difference in the mean disability score calculated from summarized scores of individual WHODAS 2.0 domains between the beginning and the end of the observation period (49.7 vs. 51.5%, *p* = 0.748).

##### Videos of tasks relating to mobility

Personal perception of mobility was contextualized through sharing of videos. In the smartphone/tablet application, patients were asked to film themselves performing basic activities. Videos were uploaded by patients showing themselves taking hold of a cup of tea or performing a similar task (27 of 30 patients) and walking five steps or an equivalent task (23 of 30 patients). All patients experienced at least some degree of movement restriction, both at the beginning and at the end of the observation period.

### Secondary analyses

The secondary analyses were based on data from study stages 1 and 3, including 60 patient interviews (one opening interview at the beginning of the observation period and one closing interview at the end).

#### Domains related to everyday life experiences in PSS

**Figure 7:**
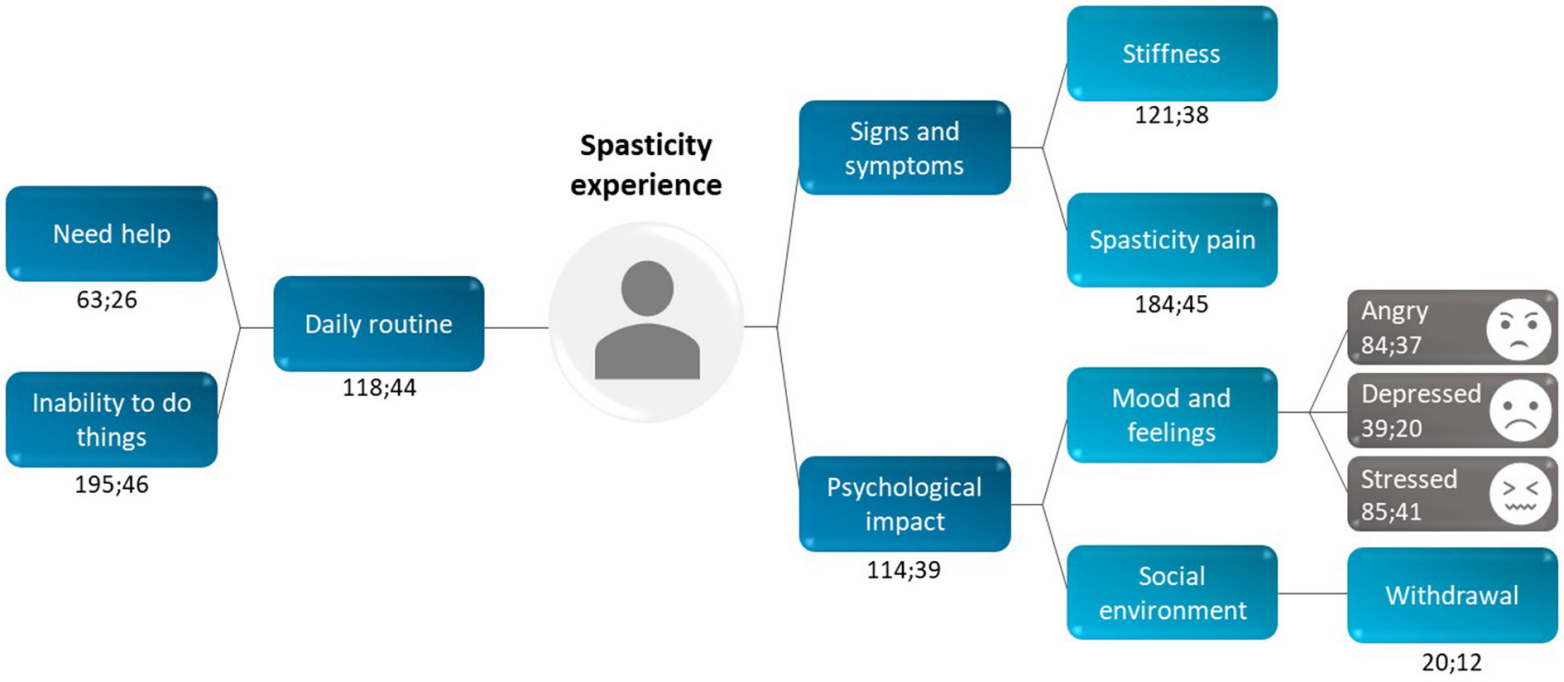
Patient perception of the overall spasticity experience: data from two interviews (stage 1 and stage 3). Each recurring theme was coded using the following format: x;y, in which x = “total number of times a theme was mentioned” and y = “number of interviews in which the theme was mentioned.” For example, the code for stiffness (121;38) was mentioned 121 times across 38 interviews.

Outputs from two in-depth interviews were used to create a mind map of the overall spasticity experience (Figure 7). This mind map consisted of frequently occurring themes; the most frequently mentioned theme was the inability to perform activities (mentioned 195 times in 46 interviews), for example: *I guess just everyday activities, like getting dressed, putting makeup on, taking a shower. Everything takes me three times as long (patient, USA)*.

The psychological and social impact of treatment was explored, with a clear correlation between spasticity symptoms and mood: *if the symptoms are worse, they would make my mood depressed more (patient, USA)*. Patients reported feeling frustrated, nervous and/or stressed, angry and/or upset, and bad or sad. Notably, patients found it hard to differentiate between the impact of the stroke and the spasticity, because both had a significant impact on their emotional state. Some patients even considered the stroke to have changed their personality: *yes, ultimately it must have changed my personality, deep inside, in terms of psychology, regarding the way to look at things (patient, France)*.

Investigating the social and professional environment revealed that patients not only have to deal with their own emotional response to their spasticity, but the reactions of those around them. In some instances, this undermined relationships. As one patient stated: *I am now on bad terms with my sister because of her reactions (patient, France)*. Others found themselves withdrawing and becoming increasingly isolated from social groups and, in some circumstances, unable to continue with their work.

All patients started BoNT-A treatment several months after their stroke and had been receiving therapy for a few years before their interview: 40.0% for over 6 years, 36.7% for between 6 and 3 years, and 23.3% for fewer than 3 years.

Receiving BoNT-A injections improved patients' symptoms (Table 2), with patients reporting less stiffness, less pain, and improved sleep. Many interviewees described themselves as “satisfied,” and/or used positive language indicating clear progress: *I feel better now, now I've been prescribed the Botox (patient, UK)*.

**Table 2:** Frequently recurring themes identified during the interviews (stages 1 and 3 of the study).

**Interpretative overarching theme**	**Category**	**Theme codes (x;y)** **(x = total number of occurrences of a theme, y = number of interviews containing the theme)**	**Example quote from the interviews**
Everyday life experience of spasticity	Perception of the condition and signs, symptoms, and impact on daily life	Spasticity pain (184;45)	“I have pain, which makes it difficult for me to move” (patient from France)
		Stiffness (121;38)	“Because the arm starts to get stiff and some pain starts, so I can't wait for this suffering to end” (patient from Italy)
		Spasms (69;20)	“The constant moving, like constant spasms, which obviously causes pain, which drains you” (patient from the UK)
	Psycho-social impact – mood, emotions, and social environment	Psychological impact (114;39)	“Generally, I've been quite depressed actually, feeling like really overwhelmed and just fed up with my hand not working” (patient from the UK)
Treatment experience	Relationship with HCPs	Physician (198;53)	“Am I going to get any better, is it going to improve any more, or have I plateaued or what? What is happening? And no-one can answer that question” (patient from the UK)
	Perception of BoNT-A treatment	Effectiveness (442;59)	“Since I started the botulinum toxin therapy, I've been able to lift my arm better. Well, my shoulder would get dislocated a lot; now it gets less dislocated. […] As for my leg, I find I can lift my knee better” (patient from France)
	Treatment satisfaction	Treatment satisfaction (25; 33)	“I'm very satisfied because I no longer feel the pain in my joints – much less anyway. I can move more or less too” (patient from the USA)
	Perception of the experience between injections	Frequency of injection (228;56)	“Very satisfied. I think they do help a lot. I just wish they were more frequent” (patient from the USA)

BoNT-A, botulinum neurotoxin A; HCP, healthcare professional.

**Figure 8:**
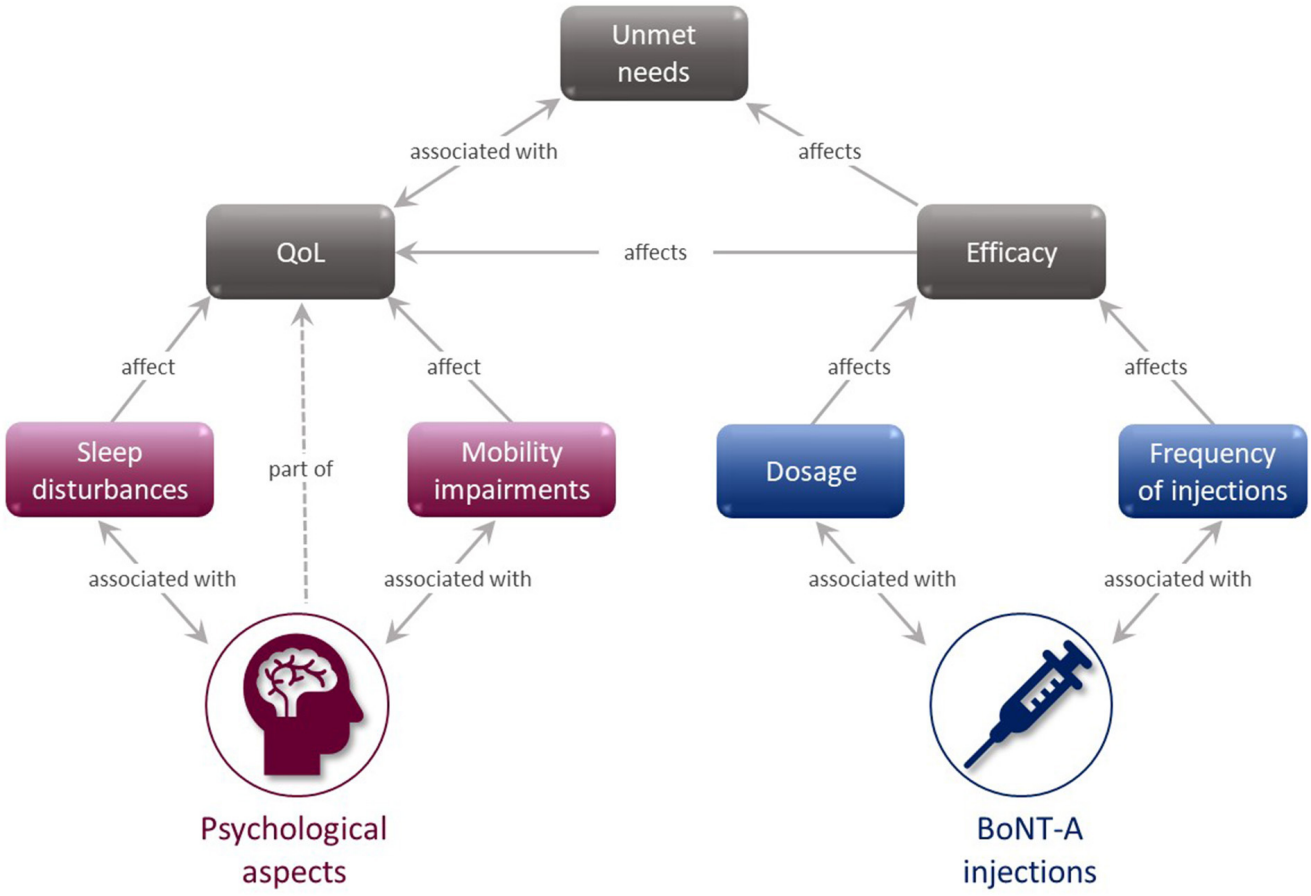
BoNT-A injection experience mind map. BoNT-A, botulinum neurotoxin A; QoL, quality of life.

Approximately 83% of patients were satisfied with their BoNT-A treatment and actively mentioned that they could feel its effectiveness rising until the second month of the treatment cycle, after which they felt it diminishing. Adapting the timing of injections to the patients' changing needs, such as seasonality or emerging symptoms, was a common desire among patients. Commentary on treatment experience was greatly affected by various overlapping factors (Figure 8), such that although a patient might think that the medication itself was of value, their overall experience was undermined by the infrequency of injection and insufficient dose. Two patient statements summarize this experience.

- *I'm the only person who knows how tight I'm getting. They're just going by reviews and studies whatever that you can only get it every 12 weeks. But my body definitely needs it more than that (patient, UK)*.
- *The ideal would be to be able to schedule the appointment as soon as there are external signs that the toxin effects are weakening... (patient, France)*.

Perceptions of surgery for spasticity were ambiguous, with patients questioning the associated risks. One patient expressed the uncertainty of a surgical outcome as it had been presented to them: *they're saying that the surgery will either work or it will go the opposite way and I'll lose all power in my arm (patient, UK)*.

Most patients reported that they benefited from the combination of BoNT-A injections and physiotherapy. However, others experienced pain, which appeared to be related to the limited experience of physiotherapists in managing spasticity. As one patient commented: *it's just like one time he didn't know what to do, and he pulled out an old textbook (patient, USA)*.

#### Relationship with leading physician

Discussions with physicians usually focused on understanding PSS and its potential course. Only 10% of patients actively stated that they had a trusting relationship with their physician and thought that their needs were considered. Others stated challenges, such as the physician's lack of didactic skills: *when physicians talk to you, they sometimes forget you're not a physician yourself (patient, France)*. The patients also commented that they felt “left alone” and/or had a lack of understanding of how their condition might evolve. One possible explanation for limitations in communication was given by a UK patient, who observed that *no doctor really knew exactly what it was, so they didn't know how to treat it either*.

## Discussion

To our knowledge, this is the first ethnographic study in patients living with PSS who were treated with BoNT-A. The study mixed quantitative and qualitative approaches to allow more granular data to be gathered than would be generated with traditional methods. This approach was chosen to capture patients' experiences of a full BoNT-A treatment cycle, including the onset, peak, and trough of treatment effects, in the context of their daily life.

In the present study, patients rated stiffness as the most burdensome symptom; this symptom showed clear improvement with treatment but overall started to return to near baseline levels at approximately 9–11 weeks after injection and some symptoms worsened at the end of observation compared to baseline. As the baseline assessments took place within 1 week post injection, it is possible that some patients had already experienced BoNT-A effects, thus not reflecting the true baseline level. Mobility decreased after Weeks 4–6 until the end of the observation period, in line with the waning effect of BoNT-A treatment. In contrast, treatment had little impact on the patients' ability to undertake activities that they would normally enjoy; arguably, increasing the use of concomitant medication might support patients in carrying out such activities.

Qualitative analysis of patients' answers indicated that they were generally satisfied with BoNT-A treatment but wished for better symptom control between the two injections. Notably, only 10% of patients described their relationship with their physician as trusting, which highlights the need to improve physician–patient communication in PSS.

The data from the current study suggest that recurrence of spasticity symptoms between injections is associated with a significant daily burden, and that the effects of BoNT-A treatment peak during the 4–6-week period post-injection and then diminished at approximately 9–11 weeks after injection; muscle spasms and difficulties in moving freely and undertaking activities increased with time. These findings are in agreement with the results from international, survey-based studies conducted in patients with spasticity, and may reflect a practice of under-dosing (7, 8). In this study, once symptoms reemerged, they continued until the end of the observation period, which may explain the increased use of painkillers and muscle relaxants over time.

The worsening of symptoms was also associated with a decrease in patient satisfaction; this was reflected in terms of overall mood. The overall burden of recurring symptoms might be associated with the need to adapt working schedule, stop working altogether and/or change job, and/or increase social restrictions. Recurring symptoms might also be linked to a higher risk of side effects due to self-medication (18, 19).

Temporal changes measured by the EQ-5D-5L and WHODAS 2.0 did not seem to be significant. This may potentially reflect a study limitation, since these measurement tools were not specifically designed to detect spasticity-related changes in health-related QoL and, consequently, they may lack the sensitivity to fully capture these changes. The first ever spasticity-related QoL tool (SQOL6D) had not been published when this study was designed and conducted, although it has subsequently been validated and disseminated for use (20, 21). Other limitations included the small sample size, a problem that is inherent to qualitative methodology, and also the relatively young age of the patients included in our study, which is not representative of the overall population of patients with PSS but which reflects the relatively greater willingness of this age group to engage in a digital survey. Patients included in the study came from different country populations, received different BoNT-A formulations (doses and injection patterns were not recorded), and had different concomitant treatments that may have contributed to the impact on their spasticity-related symptoms. This heterogenous patient profile, which displayed a variety of socio-demographic characteristics and disease experiences, may have limited the quantitative analysis but was required in the qualitative, ethnographic analysis.

Another distinctive characteristic of this study population is that patients received their PSS treatment up to a few months after their stroke, which is not the usual practice as reflected in the literature (12, 22). An increasing body of evidence suggests that there may be clinical benefits to identifying factors predictive of PSS with an aim to initiate therapeutic strategies early in the treatment pathway (22).

Finally, the timing of the injection relative to study commencement may be relevant; baseline observations were taken in the first week after injection, so some patients may have been experiencing the effects of BoNT-A treatment at the beginning of the observation period. This is supported by a recent study showing that the average time to onset of BoNT-A effect was 6.7 ± 5 days (23).

The ethnographic approach used in this study complements traditional research methods and so may allow for improved identification of patients' unmet needs. Furthermore, novel methods (such as virtually supported ethnography to gather patients' insights) may contribute to the development of patient-centric and patient-informed treatment approaches in PSS. Similar studies on a larger population may provide additional insights into the daily burden of PSS.

The findings of our research confirm previous observations of the diminishing effectiveness of BoNT-A injections between treatment sessions and show an overall high and continuous burden of PSS. This highlights the need for agents with a longer duration of action or a more flexible treatment pattern that allows for more frequent injections.

## Contribution to the field

After a stroke, patients often experience limb stiffness (spasticity), leading to difficulties with moving and taking care of themselves. The REBOT study included 30 patients who had limb spasticity after a stroke and received a botulinum toxin injection, which is used to relax stiff muscles. To understand how patients felt between two botulinum toxin injections, researchers used telephone interviews, medical questionnaires, and a smartphone application that patients used to report their symptoms, mood and any treatment effects. Patients reported that spasticity impacted their physical and mental wellbeing. The most common symptoms were arm or leg stiffness, pain, and poor sleep. Patients felt the greatest improvements in symptoms 4-6 weeks after their injection; after that period, they felt their symptoms slowly coming back. Patients wished for better symptom control, and that they could get botulinum toxin injections when needed. These results show that, although botulinum toxin is normally given every 12 weeks, some patients may need a personalized injection plan. Additionally, patients would like to be more involved with their clinicians when deciding the goals and timings for their treatment.

## Data availability statement

The raw data supporting the conclusions of this article will be made available by the authors, without undue reservation.

## Ethics statement

The studies involving human participants were reviewed and approved by an independent Institutional Review Board in the U.S. This study is non-interventional, and therefore falls outside the scope of the European Union (EU) Directive 2001/20/EC and the EU Directive 2005/28/EC. This study complies with the EU Directive 95/46/EC of the European Parliament, and of the Council of 18 May 2019 on the protection of individuals with regard to the processing of personal data and on the free movement of such data. The patients/participants provided their written informed consent to participate in this study.

## Author contributions

JJ is the primary author of this manuscript. AL, ML, and FCG contributed equally. All authors have contributed substantially to the research design, analysis, interpretation of data, and drafting of the paper and its revisions, and have approved the submitted and final version of the manuscript.

## Funding

The study and medical writing support for this paper were sponsored by Ipsen.

## Conflict of interest

Author JJ received consultancy fees from Ipsen. Authors AL and FC-G are employees of Ipsen. The remaining authors declare that the research was conducted in the absence of any commercial or financial relationships that could be construed as a potential conflict of interest.

## Publisher's note

All claims expressed in this article are solely those of the authors and do not necessarily represent those of their affiliated organizations, or those of the publisher, the editors and the reviewers. Any product that may be evaluated in this article, or claim that may be made by its manufacturer, is not guaranteed or endorsed by the publisher.
