# Supplementary material for: Experiences of patients with poststroke spasticity throughout a botulinum toxin treatment cycle: Results from a prospective ethnographic study
Source: Front Neurol. 2022 Aug 23;13:946500. doi: 10.3389/fneur.2022.946500 (PMC9479670; doi:10.3389/fneur.2022.946500)
Supplement: Supplementary file 1 [file Data_Sheet_1.docx]

# *Supplementary Material*

## Examples of Materials Shared by Patients

### Example screenshots from videos, representing patients’ attempts to perform everyday activities

#### Walking at the beginning of the observation period

|  |  |
| --- | --- |
| Example 1. Patient describing an improved ability to move their leg 10 days after  BoNT-A injection.  BoNT-A, botulinum neurotoxin A. | Example 2. Patient with some difficulty walking, moving sideways for stability 3 weeks after injection. |

#### Walking at the end of the observation period

|  |
| --- |
| Example. Patient showing difficulty with walking and balance at week 14. |

#### Holding a mug at the beginning of the observation period

|  |  |
| --- | --- |
| Example 1. Patient demonstrating difficulties with holding a mug. This patient described their frustration with not being able to lift objects. | Example 2. Patient, in an upright position, being able to drink from a mug. |

#### Holding a mug at the end of the observation period

|  | 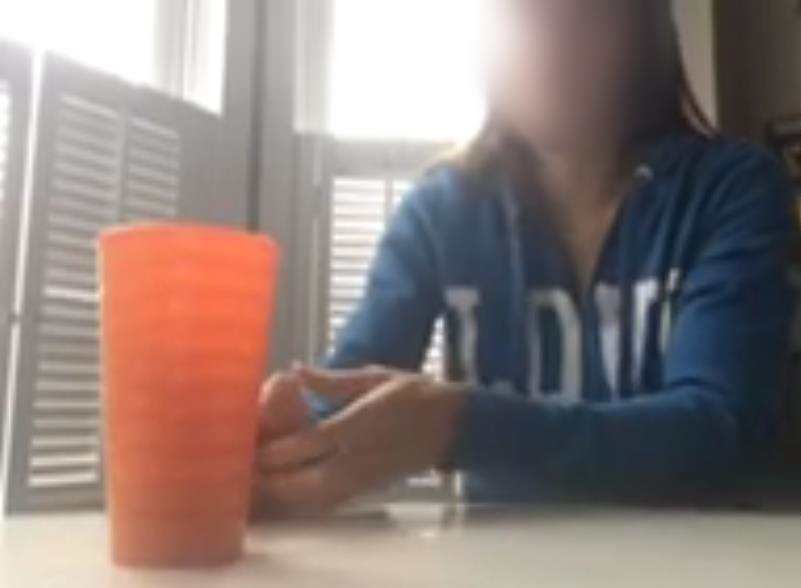 |
| --- | --- |
| Example 1. Patient describing not being able to open their hand and grab a mug. They explain that the BoNT-A injection did not improve their dexterity during a 12-week period. BoNT-A, botulinum neurotoxin A. | Example 2. Patient describes their increasing difficulty in holding a cup. At the end of the observation period, they needed their other hand to support their spastic hand while lifting the cup. |

### Example images to illustrate the highlights and low points of a week

#### Highlights and low points at the beginning of the observation period

| 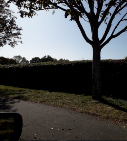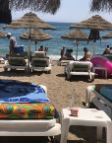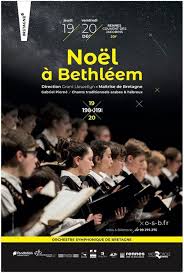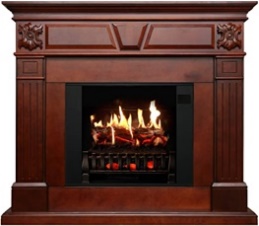 | 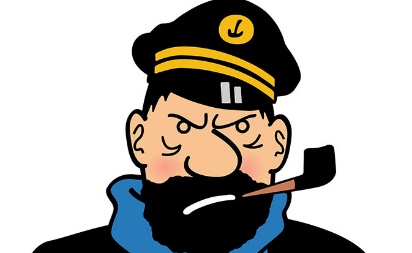 |
| --- | --- |
| Images representing the highlights of the week. | Image representing the low points of the week. |

#### Highlights and low points at the end of the observation period

| 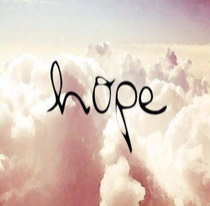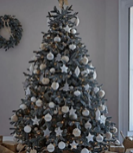 | 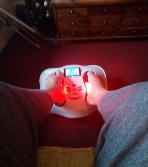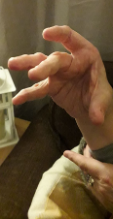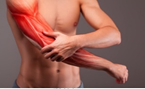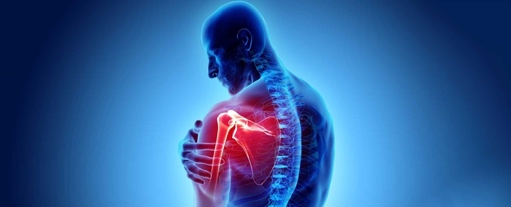 |
| --- | --- |
| Images representing the highlights of the week. | Images representing the low points of the week. |

## Statistical analyses

**Mean difference in symptoms / impairment between the beginning and the end of the observation**

| Symptoms/Impairment  (n=28) |  | P value* |
| --- | --- | --- |
| 1. Stiffness in limbs / body (mean, SD) | 0.21 (1.34) | 0.517 |
| 2. Muscle spasms (mean, SD) | 0.82 (1.28) | 0.033 |
| 3. Falling getting up from a chair, walking or bending over (mean, SD) | 0.29 (1.36) | 0.541 |
| 4. Difficulties moving freely compared to past weeks abilities (mean, SD) | 0.61 (1.55) | 0.125 |
| 5. Sleeping difficulties (mean, SD) | -0.07 (1.09) | 0.968 |
| 6. Difficulty to use any kind of transportation vehicle (mean, SD) | 0.14 (1.24) | 0.857 |
| 7. Difficulty to do things enjoyed normally (mean, SD) | -0.21 (1.13) | 0.542 |
| 8. Difficulty to keep a good relationship with family or partner (mean, SD) | 0.43 (1.07) | 0.240 |

*P-value corresponding to a Student’s t-test (the normality distribution of the data has been checked)

**EQ-5D-5L scores at the beginning and at the end of the observation**

| Scale:  1 – No problems  5 – Unable to perform | | At the beginning of the observation *n* =22 | At the end of the observation *n* = 22 |
| --- | --- | --- | --- |
| 1. EQ-5D-5L-mobility | Mean | 2.6 | 2.5 |
|  | Standard Deviation | 0.7 | 0.8 |
|  | 95% CI Lower | 2.2 | 2.1 |
|  | 95% CI Upper | 2.9 | 2.8 |
| 2. EQ-5D-5L-self-care | Mean | 2.3 | 2.6 |
|  | Standard Deviation | 0.8 | 1.0 |
|  | 95% CI Lower | 2.0 | 2.1 |
|  | 95% CI Upper | 2.7 | 3.0 |
| 3. EQ-5D-5L-usual activities | Mean | 2.8 | 2.7 |
|  | Standard Deviation | 0.9 | 0.9 |
|  | 95% CI Lower | 2.4 | 2.3 |
|  | 95% CI Upper | 3.2 | 3.1 |
| 4. EQ-5D-5Lpain/discomfort | Mean | 2.4 | 2.6 |
|  | Standard Deviation | 1.1 | 1.0 |
|  | 95% CI Lower | 1.9 | 2.1 |
|  | 95% CI Upper | 2.8 | 3.0 |
| 5. EQ-5D-5L- anxiety/depression | Mean | 2.1 | 2.3 |
|  | Standard Deviation | 1.0 | 0.9 |
|  | 95% CI Lower | 1.7 | 1.9 |
|  | 95% CI Upper | 2.6 | 2.7 |

**WHODAS 2.0 scores at the beginning and at the end of the observation**

| Scale:  0 – No difficulty  4 – Extreme difficulty | | At the beginning of the observation n = 22 | At the end of the observation  N=24 |
| --- | --- | --- | --- |
| 1. Taking care of your household responsibilities | Mean | 2.5 | 2.5 |
|  | Standard Deviation | 0.9 | 0.7 |
|  | 95% CI Lower | 2.1 | 2.2 |
|  | 95% CI Upper | 2.9 | 2.8 |
| 2. Community activities | Mean | 2.4 | 2.3 |
|  | Standard Deviation | 1.4 | 1.2 |
|  | 95% CI Lower | 1.8 | 1.8 |
|  | 95% CI Upper | 3.1 | 2.8 |
| 3. Emotional wellbeing | Mean | 2.4 | 2.5 |
|  | Standard Deviation | 1.1 | 1.2 |
|  | 95% CI Lower | 1.9 | 2.0 |
|  | 95% CI Upper | 2.9 | 3.0 |
| 4. Walking a long distance (e.g., a kilometer) | Mean | 2.6 | 2.7 |
|  | Standard Deviation | 1.4 | 1.4 |
|  | 95% CI Lower | 1.9 | 2.1 |
|  | 95% CI Upper | 3.2 | 3.3 |
| 5, Washing the whole body | Mean | 1.7 | 2.3 |
|  | Standard Deviation | 1.3 | 1.4 |
|  | 95% CI Lower | 1.1 | 1.7 |
|  | 95% CI Upper | 2.3 | 2.9 |
| 6. Getting dressed | Mean | 1.8 | 2.0 |
|  | Standard Deviation | 1.1 | 1.1 |
|  | 95% CI Lower | 1.4 | 1.6 |
|  | 95% CI Upper | 2.3 | 2.5 |
